# Comprehensive profiling and characterization of cellular microRNAs in response to coxsackievirus A10 infection in bronchial epithelial cells

**DOI:** 10.1186/s12985-022-01852-9

**Published:** 2022-07-21

**Authors:** Yajie Hu, Lan Wang, Mingmei Zhong, Wei Zhao, Yujue Wang, Jie Song, Yunhui Zhang

**Affiliations:** 1 Department of Pulmonary and Critical Care Medicine, The First People’s Hospital of Yunnan Province, Kunming, China; 2 The Affiliated Hospital of Kunming University of Science and Technology, Kunming, Yunnan China; 3 Department of Anesthesiology, The First People’s Hospital of Yunnan Province, Kunming, China; 4 Institute of Medical Biology, Yunnan Key Laboratory of Vaccine Research and Development On Severe Infectious Diseases, Chinese Academy of Medical Science and Peking Union Medical College, Kunming, China

**Keywords:** Hand, foot, and mouth disease (HFMD), Coxsackievirus A10 (CV-A10), MicroRNAs (miRNAs), High-throughput sequencing, Bioinformatics analysis

## Abstract

Coxsackievirus A10 (CV-A10), the causative agent of hand, foot, and mouth disease (HFMD), caused a series of outbreaks in recent years and often leads to neurological impairment, but a clear understanding of the disease pathogenesis and host response remains elusive. Cellular microRNAs (miRNAs), a large family of non-coding RNA molecules, have been reported to be key regulators in viral pathogenesis and virus-host interactions. However, the role of host cellular miRNAs defensing against CV-A10 infection is still obscure. To address this issue, we systematically analyzed miRNA expression profiles in CV-A10-infected 16HBE cells by high-throughput sequencing methods in this study. It allowed us to successfully identify 312 and 278 miRNAs with differential expression at 12 h and 24 h post-CV-A10 infection, respectively. Among these, 4 miRNAs and their target genes were analyzed by RT-qPCR, which confirmed the sequencing data. Gene target prediction and enrichment analysis revealed that the predicted targets of these miRNAs were significantly enriched in numerous cellular processes, especially in regulation of basic physical process, host immune response and neurological impairment. And the integrated network was built to further indicate the regulatory roles of miRNAs in host-CV-A10 interactions. Consequently, our findings could provide a beneficial basis for further studies on the regulatory roles of miRNAs relevant to the host immune responses and neuropathogenesis caused by CV-A10 infection.

**Supplementary Information:**

The online version contains supplementary material available at 10.1186/s12985-022-01852-9.

## Introduction

Hand, foot, and mouth disease (HFMD) is a common disease characterized by fever, oral ulcers, and skin manifestations affecting the palms, soles, and buttocks [1, 2]. Although HFMD is classically a mild disease, outbreaks in Asia have been associated with a high incidence of fatal cardiopulmonary and neurologic complications. Historically, enterovirus 71 (EV-A71) and coxsackievirus A16 (CV-A16) are the most common cause of HFMD [3]. However, recent epidemiological data indicated that infections with coxsackievirus A6 (CV-A6) and coxsackievirus A10 (CV-A10) have markedly increased worldwide [4, 5]. Moreover, CV-A10 infection has been often reported to cause severe HFMD and causes symptoms of fever and vesicles on other parts of the body (limbs, buttocks, and trunk), limb shaking, severe nervous system disease (aseptic meningitis and viral meningitis) [5, 6]. Currently, there is no pharmacological intervention or vaccine available for HFMD [7]. Although three inactivated monovalent EV-A71 vaccines have been licensed in China and proved these vaccines had high efficacy against EVA71-associated HFMD [8], but they did not confer cross protection for HFMD caused by non-EV-A71 enteroviruses, including CV-A10 [7, 9]. Therefore, it is urgent to investigate infection mechanism of CV-A10 for develop monovalent or multivalent CV-A10 vaccines.

MicroRNAs (miRNAs), a major class of small endogenous, noncoding RNAs, approximately 20 ~ 25 nt long, are RNA-sequence-specific post-transcriptional regulators of gene expression [10]. They are powerful regulators of various cellular activities including cell growth, differentiation, development, and apoptosis. Accumulating evidence has been indicated that miRNAs play a pivotal role in many viral infections, with different viral families expressing their own miRNAs, manipulating host miRNA expression, or showing direct or indirect regulation by host or viral miRNAs [11]. For example, miR-125b-5p is significantly increased in Japanese encephalitis virus (JEV)-infected cells and it directly targets both viral and host sequences, suggesting its role in coordinating viral replication and host antiviral responses [12]. Additionally, miR-133a is found to regulate dengue virus (DENV) replication possibly through the modulation of a host factor such as PTB [13]. Furthermore, miR-30e-3p inhibits influenza B virus replication by targeting viral NA and NP genes [14]. Thus, the above studies demonstrated that miRNAs are essential regulators of gene expression in humans and can control pathogenesis and host-virus interactions [11, 15]. Nevertheless, a large number of studies have actually investigated the impact of enterovirus infections on the cellular miRNA expression and these studies have further discussed the role of host miRNAs in enterovirus pathogenesis [16, 17]. For instance, differentially expressed miRNAs were found to involve in regulating the pathogenesis of coxsackievirus B3 (CV-B3)-induced viral myocarditis [18]. EV-A71-induced miR-494-3p impacts PI3K/Akt signaling pathway by targeting PTEN, which directly promotes EV-A71 replication [19]. CV-A16 penetrates the blood–brain barrier and then enter the central nervous system (CNS) by downregulating miR-1303, which disrupts junctional complexes by directly regulating MMP9 and ultimately causing pathological CNS changes [20]. Moreover, our previous articles have also analyzed the expression patterns of miRNAs in peripheral blood mononuclear cells (PBMC) of rhesus monkey [21], bronchial epithelial cells (16HBE) [22, 23] and human umbilical vein endothelial cells (HUVECs) [24, 25] after EV-A71 and CV-A16 infections, which clarified the regulatory roles of miRNAs in cellular processes of EV-A71 and CV-A16 infections, including immune escape, apoptosis, signal transduction, shutdown of host protein synthesis and viral replication, etc. Hence, these researches implied that dysregulated host miRNAs may directly contribute to viral pathogenesis, and in-depth research into cell-encoded miRNA could further augment our understanding of host-virus interplay for enterovirus infection, meanwhile reveal novel strategies for antiviral therapies. However, the underlying functions of miRNAs in CV-A10 infection has not been reported.

Previous studies have verified that HFMD is widely spread by fecal–oral, oral-oral, and respiratory droplet contact, thereby intestinal epithelial cells and respiratory tract epithelial cells are major target cells for enterovirus infections, and meanwhile it is believed that enteroviruses first replicate in the bowel or oropharyngeal cavity the and then trigger the subsequent symptoms [26]. Additionally, our team also found that EV-A71 infection presented more typical pathologic changes in the respiratory tract than in the alimentary tract [27]. Thence, in the present study, we aimed to adopt 16HBE cells, the pivotal target of enterovirus infection within the respiratory tract, for CV-A10 infection to investigate alterations in miRNAs due to CV-A10 infection and further analyze the functions of these changed miRNAs by sequencing technology, which might offer future perspectives regarding the mechanisms underlying CV-A10 pathogenesis.

## Materials and methods

### Virus and cell culture

16HBE cells, purchased from Jennino Biological Technology (Shanghai, China), were seeded into 6-well sterile plastic culture plates at a density of 5 × 10^5^ cells per well with Dulbecco’s modified Eagle medium (DMEM; Corning, USA) with the addition of 10% Fetal Bovine Serum (FBS; Corning, USA), 100 units/mL penicillin, 100 μg/mL streptomycin and 2 mM L-glutamine in 5% carbon dioxide (CO_2_) saturated humidified incubator at 37 °C. When the monolayers were approximately 80% confluent, DMEM was removed from each well and then was inoculated with coxsackievirus A10 (CV-A10; sub-genotype C, GenBank: MN557275), which isolated from an epidemic in Xiangyang, China, in 2017, at a multiplicity of infection (MOI) of 1. After the incubation at 0, 12 and 24 hpi, the cells were rinsed with serum-free DMEM, which was followed by the application of three freeze–thaw cycles. Cells were scraped into 2 ml of phosphate-buffered saline (PBS), and stored at − 80 °C until use. Cells infected with CV-A10 at 0 hpi were used as control.

### Virus titration in Vero cell by 50% cell culture infectious dose (CCID50) assay

Virus titers were determined using the median end point of the cell culture’s infectious dose (CCID_50_). Serially-diluted viruses were added to Vero cells grown in 96-well plates, and 8 replicate samples were used for each dilution. The 96-well plates were incubated for 7 days at 37 °C, and the CCID_50_ values were measured by counting infected Vero cell culture wells with obvious cytopathic effects and calculated by the Reed-Muench method.

### Immunofluorescence (IF) microscopy

16HBE cells were seeded onto poly-L-lysine-coated coverslips (Solarbio, China) and treated as previously described. At the indicated time, the cells were washed in PBS, fixed with 4% PFA (Solarbio, China) and permeabilized with 1% Triton X-100 in PBS. The cells were blocked with 5% BSA at room temperature for 1 h and then incubated with the primary antibodies in blocking solution against CV-A10-VP1 (1:1,000, Genetex, China) overnight at 4 °C. Next, cells were washed with PBS three times and then incubated with Alexa Fluor 647-conjugated donkey anti-mouse IgG (Millipore, USA) for 1 h at room temperature. The nuclei were counterstained with 4′, 6-diamidino-2-phenylindole (DAPI, 1:4,000, Beyotime, China). Slides were mounted with antifade reagent (Solarbio, China) and observed using a laser scanning confocal microscope (Leica, Germany). The images were captured and processed using Adobe Photoshop 7.0 software.

### RNA extraction, construction of small RNA libraries and deep sequencing

Three replicates of CV-A10-infected cells at different time were mixed together and used for RNA extraction. Briefly, total RNA from the harvested cells was extracted with the Trizol reagent (Invitrogen, USA). The concentrations of total RNA of samples were determined by NanoDrop 2000, and the RNA integrity and purity was assessed using an Agilent Bioanalyzer 2100. All RNA integrity numbers (RINs) for the samples were > 7.0 and rRNA 28S/18S ≥ 1.6, indicating that the RNA was of good quality and suitable for constructing small RNA libraries.

Subsequently, the qualified RNA samples were fractionated by 15% denaturing polyacrylamide gel electrophoresis (PAGE), and then small RNA fragments between 18 and 35 nt in length were isolated from the gel. The sRNA molecules were ligated to a 5’ adaptor and a 3’ adaptor by T4 RNA ligase (Promega, USA). Next, the adapter-ligated sRNAs were converted to cDNA by RT-PCR following the Solexa sequencing protocol (Illumina, San Diego, CA, USA). Small RNA sequencing was performed in ANOROAD Genome Inc. (Beijing, China) using a NEBNext® Multiplex Small RNA Library Prep Set for Illumina® (NEB, USA) on the Illumina HiSeq™ 2500 platform.

### Computational analysis of sequencing data

#### Sequencing data processing

Raw sequences usually include contaminants and low-quality reads. The raw reads were processed to filter the reads that contained reads with 5’ primer contaminants, reads without a 3’ adapter or the insert tag, reads with high poly A or T, and low-quality reads (Q30 < 90%), resulting in clean reads. Then, the annotation process of sequences 18–35 nt long from the clean reads was performed as follows: (1) The small RNA tags were first mapped to the reference sequence using Bowtie, without allowing any mismatches, to analyze their expression and distribution relative to the reference sequence; (2) Next, the mapped small RNA tags used to search for known miRNAs were aligned against the miRBase 21.0 database (http://www.mirbase.org/) with ≤ 1 mismatches; (3) The mapped small RNA tags were also mapped to Rfam, RepeatMasker, to annotate the tags and remove those originating from protein-coding genes, repeat sequences, ribosomal RNAs (rRNAs), transfer RNAs (tRNAs), small nuclear RNAs (snRNAs), and small nucleolar RNAs (snoRNAs); (4) The remaining unannotated tags were used to predict novel miRNAs by the miReap program (http://sourceforge.net/projects/mireap/).

#### Global miRNA expression profiling

All raw counts of miRNA reads were further normalized by transcripts per million reads (TPM) using the following calculation: normalized expression = mapped read count/total mapped reads × 1,000,000, and the miRNA expression levels between the two groups were compared using the DESeq R package. miRNAs with a *P* value < 0.05 and a fold change ≥ 2 or ≤ 0.5 were treated significantly different among the groups. All unique and shared differentially expressed miRNAs in the infected-groups were presented in a Venn diagram by using Venny 2.1 (http://bioinfogp.cnb.csic.es/tools/venny/index.html). Hierarchical cluster analysis with the shared differentially expressed miRNAs of the infected-groups was performed by Cluster 3.0 and Tree View 1.6 programs (http://rana.lbl.gov/eisen).

#### Target prediction of miRNAs

The target genes for differentially expressed miRNA were predicted using the miRanda (http://www.microrna.org/microrna/home.do), PITA (http://genie.weizmann.ac.il/pubs/mir07/mir07_dyn_data.html) and Targetscan (http://www.targetscan.org/vert_60/) algorithms. The three types of miRNA target gene prediction software have different focuses and different prediction capabilities. Thus, in this study, we decided that the consensus targets predicted by at least two programs were selected as the ultimate genes targeted by differentially expressed miRNAs for follow-up exploration. And the parameters for miRanda were set to a score higher than 155 and a free energy lower than − 20 kcal/mol, the parameters for PITA were set to a score of △△G lower than − 10, and the parameters for Targetscan were set to the top 200 genes.

#### Gene ontology (GO) and Kyoto encyclopedia of genes and genomes (KEGG) pathway analysis

In order to obtain biological information from the target genes of differentially expressed miRNAs, an enrichment analysis was performed with the Database for Annotation, Visualization, and Integrated Discovery (DAVID, https://david.ncifcrf.gov/) web tool. GO terms are produced based on their biological process (BP), molecular function (MF) and cellular component (CC), which was mainly applied to explore the distribution and potential biological functions of candidate target genes. Similarly, KEGG pathway enrichment analysis integrates genomic, chemical knowledge, and system functional information, which we used for deciphering target genes involved in significant metabolic or signal transduction pathways. The selected parameters for the study were the multiple test adjustment by Benjamini and Hochberg and the significance level set at 0.05.

#### Regulatory network construction

We performed network analysis of the intersected target genes between GO-BP-related genes and Pathway-related genes. The common target genes were furnished to GeneMANIA that incorporates large functional association data such as co-expression, colocalization, physical interactions, shared protein domains, pathway, and genetic interactions, etc. Meanwhile, the miRNAs corresponding to the intersected target genes were also identified, which were further used to construct a miRNA-mRNA regulatory network. Ultimately, the network visualization and analysis tool Cytoscape software was applied to draw the networks.

#### Validation of the differentially expressed miRNAs and their target genes by quantitative reverse transcription polymerase chain reaction (RT-qPCR)

Based on the results of the above analysis, the expression levels of the four miRNAs (namely, hsa-miR-663a, hsa-miR-145-5p, hsa-miR-455-3p, hsa-miR-940) and their target genes (namely, TGFB1, RYR1, PIK3R1, PNMA3) by RT-qPCR (Additional file 3: Table S1). In brief, total RNA was extracted using TRIzol reagent (TIANGEN, China) according to the manufacturer’s instructions and then subjected to reverse transcriptase reactions with PrimeScript™ Reverse Transcriptase Reagent Kit with gDNA Eraser (Perfect Real Time) (Takara, Japan). RT-qPCR were carried out with the SYBR Green PCR Master Mix (Takara, Japan) on a 7500 Fast Realtime PCR system (Applied Biosystems, USA) under the following thermal cycling conditions: 50 °C for 2 min, 95 °C for 10 min, followed by 40 cycles at 95 °C for 10 s and 60 °C for 30 s. The melting curve was analyzed from 60 to 95 °C at an incremental rate of 0.5 °C/10 s. The relative expression levels of miRNAs and target genes were calculated according to the equation 2^−ΔΔCT^ method using U6 and β-actin, respectively, as the internal control gene for the normalization analysis. At least three biological replicates were conducted for each individual experiment. The sequences of the specific primers used in the RT-qPCR analysis are shown in Additional file 4: Table S2.

#### Statistical analysis

For sequencing data, raw reads achieved from each library were normalized to TPM. For RT-qPCR, the experiments were tripled and data were given as mean value ± standard deviation (SD). All statistical analysis were performed with SPSS 18.0 (SPSS Inc., USA) and GraphPad Prism 5 (GraphPad Software, USA), with a *P* value < 0.05 considered statistically significant.

## Results

### CV-A10 replicate in 16HBE cells

In CV-A10-infected cells, the production of infectious virus particles showed a constant rise (Additional file 1: Fig. S1A) before 24 h and then began to decline, suggesting that the 16HBE cells were highly susceptible to CV-A10. To further address the question of whether CV-A10 possesses a more efficient replication ability to infect the epithelium, IF experiment was employed. The results shown in Additional file 1: Fig. S1B revealed that the expression of the viral structural protein VP1 of CV-A10 significantly increased with time. And it was clearly seen that the infection rate of CV-A10 at 12 h was about 50%, and that at 24 h was approximately 70%.

### Comprehensive overview of sequencing data in 16HBEs with infection of CV-A10

**Fig. 1:**
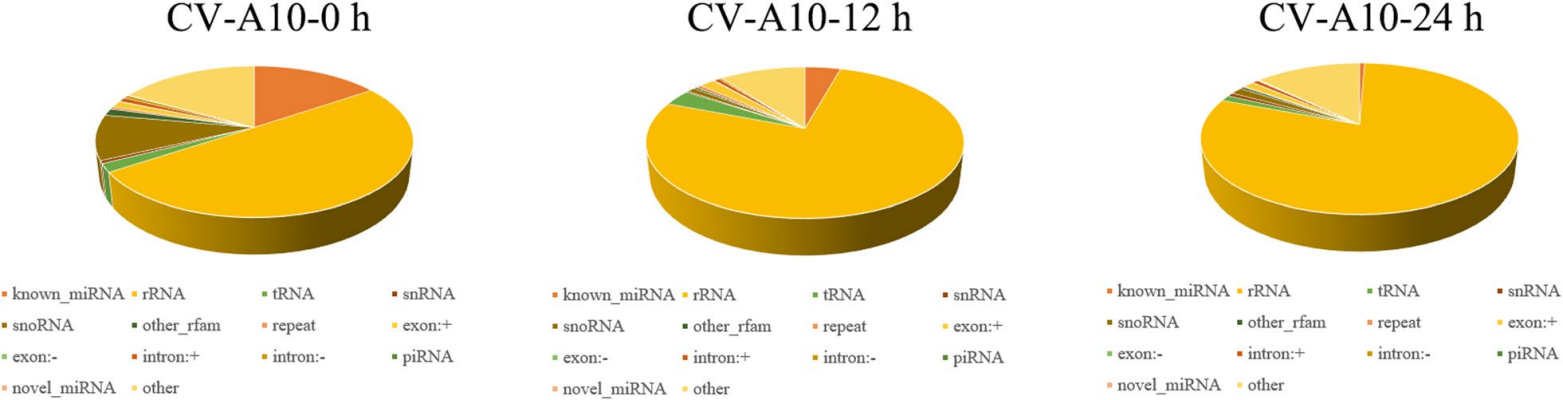
Distribution of small RNAs among different categories: infected and uninfected CV-A10 groups

To evaluate the impact of CV-A10 infection on 16HBE cells miRNAs, we performed high-throughput sRNA sequencing using the Illumina platform on sRNA libraries obtained from CV-A10-infected 16HBE cells. As shown in Table 1, a total of 83,758,020, 82,874,278 and 80,230,554 raw reads were obtained from Control, CV-A10-12 h, CV-A10-24 h groups, respectively. After removing adaptors, junk and low-quality reads, 6,307,541, 6,132,486 and 9,908,215 clean reads were identified in Control, CV-A10-12 h, CV-A10-24 h groups, respectively. Meanwhile, the Q30 values were calculated and it was demonstrated that all groups had Q30 values greater than 90%, which indicated the high quality of the sequencing of these samples. Then, the Perfect Match Reads and 1nt-mis Match Reads were analyzed, and it was found that except that the match rate of the control group was only 75.84%, the match rates of the infected groups were all over 90%. In addition, all of the clean reads were annotated and classified as known miRNA, rRNAs, tRNAs, snRNA, snoRNAs, other_rfam, repeat, exon-sense, exon-antisense, intron-sense, intron-antisense, piRNA, novel miRNAs, and other (Fig. 1). Moreover, the number of known miRNA and novel miRNAs was listed in Table 1.

**Table 1:** Details of small-RNA sequencing information

Groups	Control	CV-A10-12 h	CV-A10-24 h
Raw reads	83,758,020	82,874,278	80,230,554
Clean reads	6,307,541	6,132,486	9,908,215
Q30 value (%)	92.13	91.85	92.1
Perfect match reads	3,849,520	4,932,215	8,408,917
1nt-mis match reads	933,887	696,005	718,257
Match rate (%)	75.84	91.78	92.12
Known miRNA number	740,373	243,049	56,659
Novel miRNA number	7620	1815	12,810

### miRNA expression signature in response to CV-A10 infection in 16HBE cells

**Fig. 2:**
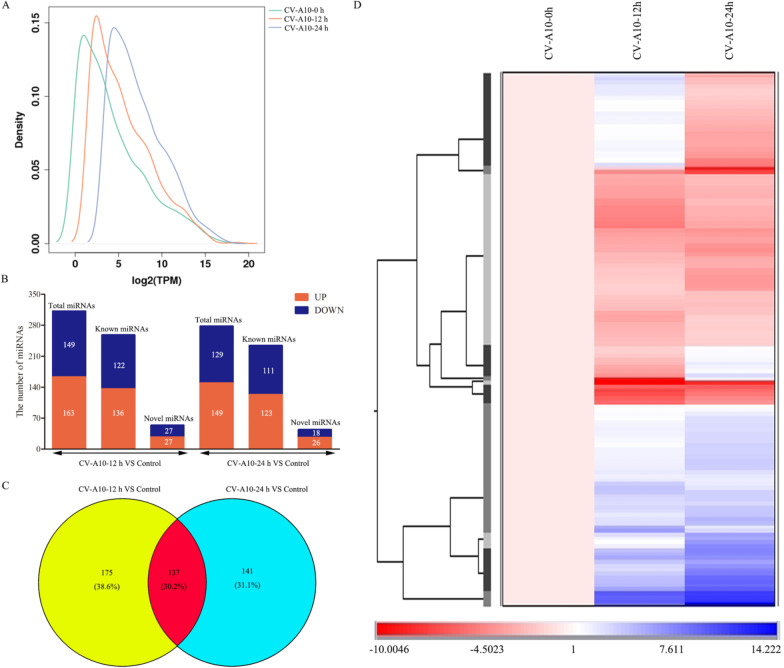
The miRNA signatures of CV-A10 infection. **A** Distribution of miRNA expression values in Control, CV-A10-12 h and CV-A10-24 h groups. **B** Bar graph showing the number of differentially expressed miRNAs in different groups. **C** Venn diagram displaying the overlapped miRNAs during the process of CV-A10 infection. **D** Distribution patterns across different samples based on common differentially expressed miRNAs analyzing by hierarchical clustering in HUVEC cells following CV-A10 infection

Overall distribution of miRNA expression values was displayed in Fig. 2A, and in order to further determine the changes in miRNA expression patterns in 16HBE cells during CV-A10 infection, global cellular miRNA expression patterns following infection were compared with those of the controls. In this study, only those differentially expressed miRNAs with a *P* value < 0.05 and a fold change ≥ 2 or ≤ 0.5 are described. The results showed that, compared with the control group, there were 136 up-regulated and 122 down-regulated known miRNAs, as well as 27 up-regulated and 27 down-regulated novel miRNAs in the CV-A10-12 h group (Fig. 2B). Additionally, there were 123 up-regulated and 111 down-regulated known miRNAs, as well as 26 up-regulated and 18 down-regulated novel miRNAs in the CV-A10-24 h group relative to the control group (Fig. 2B). Subsequently, the data for all the differentially expressed miRNAs were also graphed into a Venn diagram. Common and distinct differentially expressed miRNAs in response to CV-A10 infection at different time points were revealed. A total of 137 overlapped miRNAs was found (Fig. 2C). Simultaneously, these overlapping differentially expressed miRNAs were submitted to unsupervised hierarchical clustering to construct a heat map based on the differential expression patterns with log2 values (infected/control) and fold changes. It was clearly seen that the visual summary of the dynamic changes in the common miRNAs following CV-A10 infection at two time points (Fig. 2D). Moreover, it was also observed that the infected-samples were clustered together and separated from the control sample (Fig. 2D). Therefore, these results suggested that miRNA expression patterns induced by CV-A10 infection might be time-specific.

### Series-cluster analysis of the overlapping differentially expressed miRNAs

**Fig. 3:**
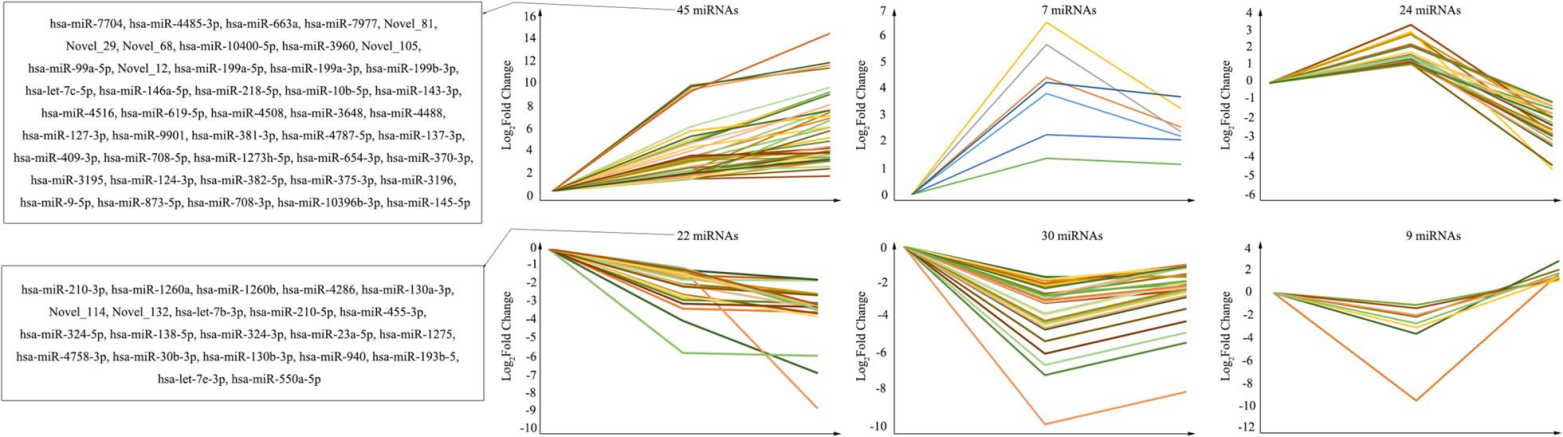
Trend analysis of differentially expressed miRNAs in response to CV-A10 infection over time

To find out the key miRNAs of interest from the 137 overlapping miRNAs, we performed a trend analysis. It was found that these miRNAs could divide into 6 expression trends: (1) Sustained up-regulation with time (45 miRNAs); (2) Both up-regulated at 12 hpi and 24 hpi, but the increasing degree at 12 hpi was lower than that at 24 hpi (7 miRNAs); (3) Up-regulated at 12 hpi, but down-regulated at 24 hpi (24 miRNAs); (4) Sustained down-regulation with time (22 miRNAs); (5) Both down-regulated at 12 hpi and 24 hpi, but the decreasing degree at 12 hpi was higher than that at 24 hpi (30 miRNAs); (6) Down-regulated at 12 hpi, but up-regulated at 24 hpi (9 miRNAs) (Fig. 3). Nevertheless, the miRNAs we focused on were those that were consistently up-regulated or down-regulated, as changes in this same persistent trend over time may be important miRNAs involved in pathological progression following CV-A10 infection. Furthermore, the typical stem-loop structures of novel differentially expressed miRNAs were displayed in Additional file 2: Fig. S2. In order to further learn the functions of these key miRNAs, the putative targets were predicted by employing the web-based software miRanda, PITA and Targetscan. The results revealed that a total of 549 target genes for 45 up-regulated differently expressed miRNAs and a total of 297 target genes for 22 down-regulated differently expressed miRNAs were successfully detected (Table 2).

**Table 2:** List of up- and down-miRNAs and their corresponding putative targets

Up-regulated miRNA	Target genes
hsa-miR-7704	GNG7, GNG3, CTD-2207O23.12, FLJ27365, SUSD2, TULP3, HSPB6, NDUFS8, TUBB4A, PPP1R27, DNAH17, CLIP3, LEF1, C8G, GDE1, FIBCD1, RTBDN, RUVBL2, GNAO1, DERL3, MT-ND4L, NINJ1, VAX1, ANKRD53
hsa-miR-4485-3p	CHMP4C, PARK7, LACTB2
hsa-miR-7977	MUC19, TRAPPC2P1, AC106017.1, PEX26, PRR13, SMIM12, KDELR1, ORAI2, C18orf32, UBC, TRAPPC2, ZNF70, C1orf210, SMUG1, TNFRSF13C, MSRB1, CD3D, APOBEC3F, PHB2, RPL24, PCBP1, SSH2, CD84, TRUB2, PRR5L, CDNF, RPL37A, RP11-47I22.3, ZNF726, NDUFA7, MRPL27, UQCR11, MRPS12, COX6B1, MAVS, TBC1D16, PCNP, C14orf2, LPAR2, CLEC17A, MTHFD1, IBA57
hsa-miR-99a-5p	EPDR1, AGO2, TTC39A, ST6GALNAC4, AP1AR
hsa-miR-218-5p	GLCE, SERP1, MOSPD1, C3orf70, TUB, VOPP1, NAPEPLD, TPD52, KCTD16, SGCZ, EIF5A2, HPGD, SAMD12, ONECUT2
hsa-miR-4516	SPATA33, WNT8B, ZBTB20, ASXL2, AL441883.1, RP11-210M15.2, SCN3B, PPP1R11, RP11-664D7.4, FBXO48, KLF8, GRIK5, GRIN2B, KSR2, PIGO, ING4, RAB19, CBL, PIP, RHO, FBXL20, RBM23, ZNF133, CCDC23, IGF2R
hsa-miR-4508	BOK, CLYBL, AKT2, ARHGAP39, PPDPF, TBX1, REPIN1, LSM7, VTN, SYT7, TMEM141, COL20A1, NFIX, VWA1, WNK2, SH3GL1, SURF4, NAPA, SLC7A5, PRX, CRHR2, ATG4B
hsa-miR-9901	THRSP
hsa-miR-409-3p	FAM229B
hsa-miR-3195	REPIN1, ZNF74, MZF1, NRG2, C8orf82, PAOX, APC2, AL591479.1, C19orf26, IFLTD1, AC103801.2, BRF1, LRRC14, C1orf229, EVX1, MT-ND4L, KIFC2, TUBB6, AL360004.1, KLHL3
hsa-miR-375-3p	RLF, POC1B, ELAVL4, COLCA2
Novel_29	–
Novel_68	–
Novel_12	–
Novel_81	Mir151b, LINC00273, RBPMS-AS1, GYG2P1, CNR2, 8-Sep, c7orf25, MIR4781, CECR2
Novel_105	SKOR2, B3GALT2, KIAA0513, MPDZ, RP1, COL19A1, SLC12A2
hsa-miR-9-5p	RTL1
hsa-miR-873-5p	MECP2, alas2, MAN2C1, c16orf62
hsa-miR-708-5p	SND1-IT1, GYG2P1, ZKSCAN8, KPNA4, FCAMR, C1orf74, POM121L2, MYO1E, GRIA4, G3BP1, TEX261, CARD14, CXCL9, SLC31A1, DRAM1, MAS1, GTF3C4, DCAF10, MDGA1, ASPA, TP53BP1, NNAT, MIR6132
hsa-miR-708-3p	DMRTC2
hsa-miR-663a	DND1, HOXA10-AS, colgalt2, RAB3B, ISLR2, KLHL21, TRIM36, TRPM2, DCTD, IDUA, TGFB1, USP14, KLHL42, OPRK1
hsa-miR-654-3p	BEAN1, AREL1
hsa-miR-619-5p	akap6, CEP250, MGA, UNC13C, MYO5A, ARFGEF3, DENND4C, FN1, AIM1, SMG1, NBEAL1, PLCE1, KALRN, URB1, DOCK9, ATM, TRPM7, TEP1, MYO10, ZNFX1
hsa-miR-4787-5p	LOC101928053, ZNF628, BCL9L, TMEM210, TMEM121, WT1, FMNL1, EP400, FZD4, UTF1, INHBB, PRMT2, ADCY8, ZNF414, AFDN, SOX9, FOXN1, MIR7855, RNF10, AP2B1
hsa-miR-4488	SEMA6C, ADAMTS8, RASD2, MADCAM1
hsa-miR-3960	SGK223, Tgfbr3l, RBM15B, TMEM158, USP51, c9orf69, LOC101928733, HPN-AS1, NEURL1B, ZNF337-AS1, NHSL2, FOXO6, LOC100128531, CNGA1, NANOS1, TUBB4B, USP7, RSBN1L, NOTUM, MANEAL, FMNL1, CNTN2, TBX1, GJD3, NKX2-5, MEX3D, GPR139, IRX5, KCMF1, SSNA1, EIF1, CEBPB, PLEKHG5, KCNG3, LONRF2, CHD3, CTNND2, GSG1L, LETM1, cenpv, C1QL3, ZNF219, OTUD1, ZNF367, UNCX, NKX6-1, RPRD2, CYGB, GUCY1A2, SETBP1, VEGFC, PCDH15, fam135b, 4-Mar, GATAD2B, ATP1B1, ZCCHC2, pelp1, BNIP2, PDX1, FOXF2, DNAJC1, ITM2B, CKAP4, BICDL1, BTF3L4, TRMT5, FAM193A, MTHFD1L, BCL11A, ARID1A, PLPPR5, PAPD7, PRDM13, PPP1R9B, BLMH, CCNY, TNKS2, EVX1, HOXA13, NOVA2, UBR5, BMF, MIB1, KIF3B, ADNP, RIMS4, SMARCB1, HCN2, RCOR1, SEPHS1, PGR, APBB1IP, DGKD, FAM50A, ARID1B, ADRB1, CLEC16A, CDK11A, CACNA2D2
hsa-miR-382-5p	LINC00301, TPST1, USP39, MED22
hsa-miR-381-3p	EHF, DUSP3
hsa-miR-370-3p	LOC101928535, PACS1, ZBTB39, ZC3H18, IL2RB, LAG3, PPP1R12A
hsa-miR-3648	GPC4
hsa-miR-3196	GLTSCR1
hsa-miR-199b-3p	PTPRF, c10orf2
hsa-miR-199a-5p	KCTD16, GOLGB1, CD5L
hsa-miR-199a-3p	PTPRF, c10orf2
hsa-miR-146a-5p	LOC101929662, ARID1A
hsa-miR-145-5p	SOCS7, LOC283887, RYR1, WWOX, wdr97, BEND3, COL4A3, RNF187, GRIP1, MYH15, SLC24A4, ARHGAP32, c10orf76, IPCEF1, cyp46a1
hsa-miR-143-3p	SPINT3
hsa-miR-137-3p	WBP1L, DR1, SSR3
hsa-miR-127-3p	MIR151A
hsa-miR-1273 h-5p	MIR4534, GRIN2B, ASB16-AS1, LOC100506321, PSMA2, LINC00847, LOC101927257, HOXB-AS1, FLJ43879, LOC729867, ZNF469, MIR28, RNPS1, TGM2, TLK1, ZKSCAN8, SVIP, GDAP2, RNF216P1, ZNF284, MRPL30, ZNF445, EWSR1, RNASE10, MXRA7, IL17RA, UMODL1, spryd4, SLC35E3, TRAPPC12, RNF150, SYAP1, SIN3A, ZNF558, cox11, METTL2B, LRPAP1, PPM1L, atad3b, TAB3, INO80C, PTGES, NXPE3, FHDC1, HRH4, RSPH3, ADGRE2, RREB1, TROVE2, MIR4781, PLEKHA3, GGCX, UMPS, NANOG, ZPR1, CLIP2, STK24, RNF125, MANBAL, EFS, PUS7, RBMS2, SCARB1, MED17, PHLPP2, CDH1, CASP10
hsa-miR-124-3p	CDK11B
hsa-miR-10b-5p	GABRB1
hsa-miR-10400-5p	DGKK, CTD-2194D22.4, CBR3-AS1, NUTM2B-AS1, FOXI3, SP5, MDM4, TSC22D2, ZBTB44, GRM7, NANOS1, ESPN, IRS2, ATP6V0A2, JAG2, BTBD6, EXT1, skida1, GAS1, GATA2, PDE4DIP, OLIG3, GRIN1, FOXG1, hsf5, ZDHHC14, OTP, XKR6, DNAJC21, CASKIN1, PRRX2, LARGE2, ZNF367, UNCX, DCLK3, PAXBP1, SPPL3, UBN2, SYN2, SCAF4, TRIP12, CABLES2, YTHDF1, ASTN2, SEMA6C, barhl2, LMO4, PCSK6, ZIC5, PHLDA1, FAM117B, GRHL1, PRR7, ZC3H4, COPB1, MGAT3, KLF2, MMP24, ETF1, IRF2BPL, EXOC8, cpeb3, UBE2R2, RIC1, CCNE1, NOVA2, CA2, CD99L2, HCN2, hivep1, KAT6A, PHLPP1, KCNN2, ATP2A3, SLK, slc9a3r2, POLD1, QSER1, CAMK2B
hsa-miR-10396b-3p	c3orf70, AMER3, SOX12, USP38, ZNF219, LILRB2, PLBD1, CSK
hsa-let-7c-5p	SPACA6P-AS, PFKFB1, DICER1

Down-regulated miRNAs	Target genes
hsa-miR-4286	PARVG, CBX2, RP11-210M15.2, WLS, CD59, FAM222B, DYNLL2, PHF1, APLN, H1F0, EMC2, PRX, PPIL1, B4GALT7, ECHDC1, TNFAIP8L3, C14orf144, C3orf62, TEX22, CTRC, ZBTB7B, FOXO4, TMSB4X, CDK9
hsa-miR-1275	PPP2R2D, KCNC3, SPRED3, NFIC, MECP2, NOVA2, CTB-54O9.9, ZBTB7A, PRKACA, VAMP2, SPA17, LUC7L, IGF2, KIR3DX1, NFIX, ID1, SCRT2, GATSL2, SDHAF1, SIRT2, APLN, HMGA1, PIN1, CEBPG, CBLN1, FOXP4, NEUROD2, SPOCK1, DIRAS2, GPBP1, PRR26, RAB11FIP5, LRRC28, SH3PXD2A, TCEB2, SLC8A2, UBTF, MSI1, ZSWIM4, HSPB6, URM1, NUDT18, HRK, EMC10, SPTAN1, DDA1, DAGLA, ACTB, FAM155B, PCDHGA12, TSPAN14, NTPCR, GRWD1, PCDHGA10, CD8B, LURAP1, RALY, MTHFR, PKNOX2, AL117190.3, CLIP3, MDK, STX8, MXD1, BZRAP1, WNT7B, IGF1, HPCA, C1orf106, PIP4K2B, CISD3, DUSP8, DOT1L, CYP2C19, ABI2, ZBED3, SPINT1, SPRR1B, ATXN1L, BCL7A, SYT7, FTO, CTSB, PIANP, KIAA0141, RP11-195F19.5, C17orf96, NKD1, C11orf42, TEKT4, MAPK4, M6PR, TBC1D7, CXADR, HSD11B2, SLC22A25, RBM23, OVOL2, UBALD1, THSD4, PURG, PTGIS, FN3K, RNASE13, MDGA1, MPZ, NDEL1, VPS37D, DDX17, TBC1D16, GATAD2B, ENY2, REEP6, KLK4, PDE4A, ZNF385B, NAPA, ZNF677, SORBS3, SLC25A42, GINS2, CNIH2, SLC25A23, RAB3A, FAM216B, CBX5, ZNF691, DOK1, RP11-368I7.4, PAX5, AGFG2, POU2F2, CD3E, CUX1, HOXC12, SLC7A8, CRYBB1, SCUBE3, FGF1, RARA, S100G, AC007405.2, C12orf75, NR1H4, RNF115, GJB4, CLDN2, FAM50B, HNRNPAB, IGF2BP1, DUSP2
Novel_132	ZNF773
Novel_114	SIVA1, CREBBP
hsa-miR-940	PNMA3, UBE2Q1
hsa-miR-550a-5p	LOC102724908, MIR550A3, MIR550A1, MIR550A2, EPM2AIP1, PCDH15, FEM1A, DHCR24, SLC44A1, SLC11A1
hsa-miR-4758-3p	LOC105370648, LOC101929054, JRK, SPANXA2, DCAF12L1, SPANXA1, SPANXD, PTCH1, HMCES, AEN, UACA, SH2B3
hsa-miR-455-3p	PIK3R1
hsa-miR-324-5p	CTNND2, TIMP1
hsa-miR-324-3p	PWRN1, FAM118B, HSF1, MAF1, ZNF556, PRNP, CLSTN1, ZNF672, TRUB2, TMEM41A, ZBTB7B, ANKRD9, HERC5, RNF121, RNF112, spdef, ZNF644, EPX, qsox1, POLD2, MYH14, HERC1, CAPN15, coro1a, DSP, CYP26A1, QSER1, SYNE2
hsa-miR-30b-3p	LOC101927460, ITSN1, FAM210A, lcmt2, ANAPC16, ADCY1, SVOPL, UBR2
hsa-miR-23a-5p	RINL, MYO1D, KSR2, ZNF577, CHRNA2, HGD, TNFSF8, TXK, CTSA
hsa-miR-210-5p	LINC00639, PRSS53
hsa-miR-210-3p	SLC25A28, ADAMTS8
hsa-miR-193b-5p	SEPHS2, OR8J3
hsa-miR-138-5p	RNF103, MUC5AC, gaa, SHISA5, PLA2G4D, CCDC3, DLX4, IPCEF1, ITIH4
hsa-miR-130b-3p	HTT, TCF4, fam47e, EIF5B, IL12RB1, EXOSC7
hsa-miR-130a-3p	TCF4, DDX17
hsa-miR-1260b	MAML2, WDR26, PHF19
hsa-miR-1260a	ngb
hsa-let-7e-3p	SPACA6P-AS
hsa-let-7b-3p	Mirlet7a1, MIRLET7A2, MIR4763, KIAA0825, AIM1

### Functional annotation and pathway enrichment analysis of predicted target genes during CV-A10 infection

**Fig. 4:**
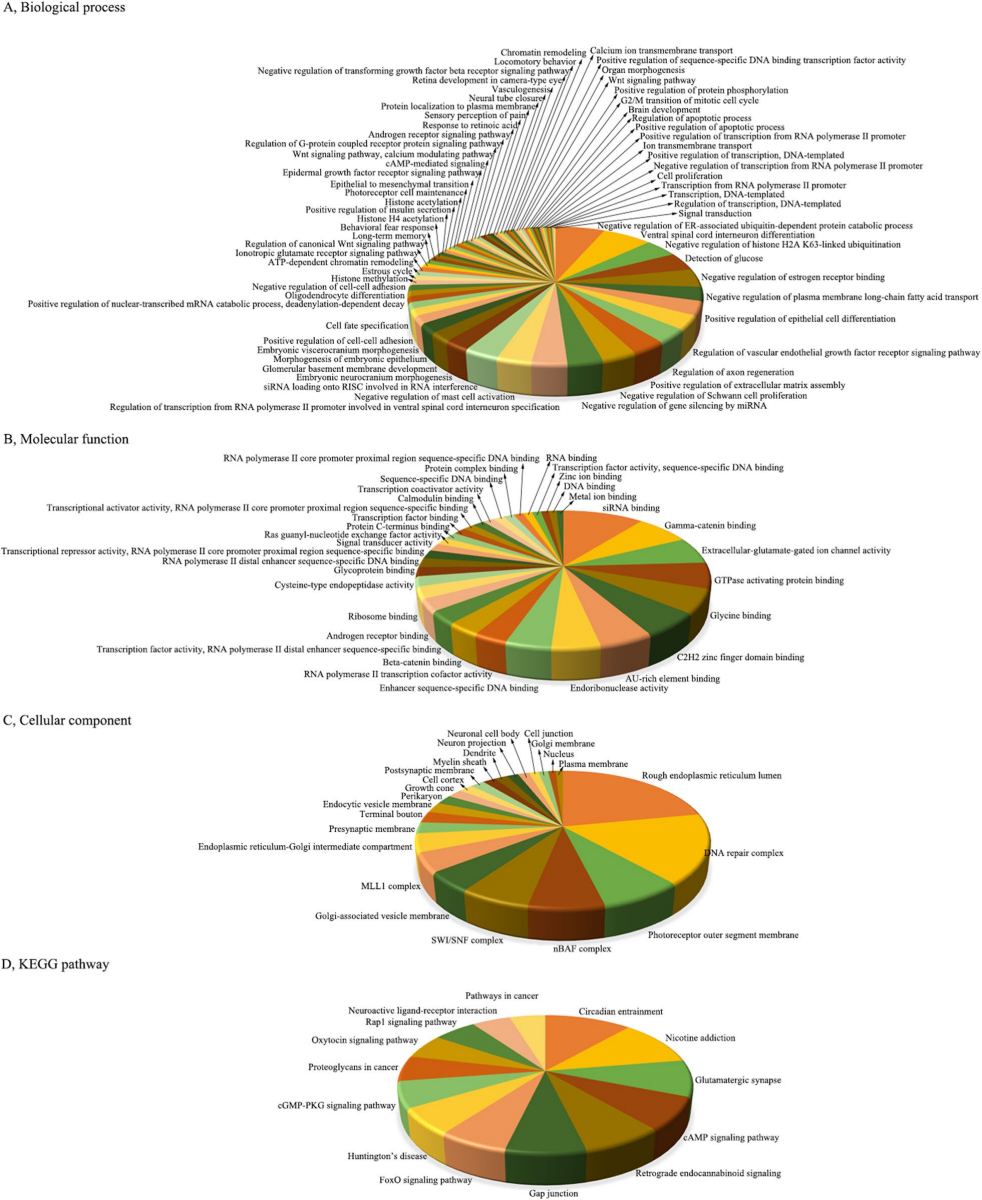
Functional enrichment analysis of target genes of persistent up-regulated miRNAs. **A** GO terms for BP of target genes. **B** GO terms for MF of target genes. **C** GO terms for CC of target genes. **D** KEGG Pathway annotations for target genes

**Fig. 5:**
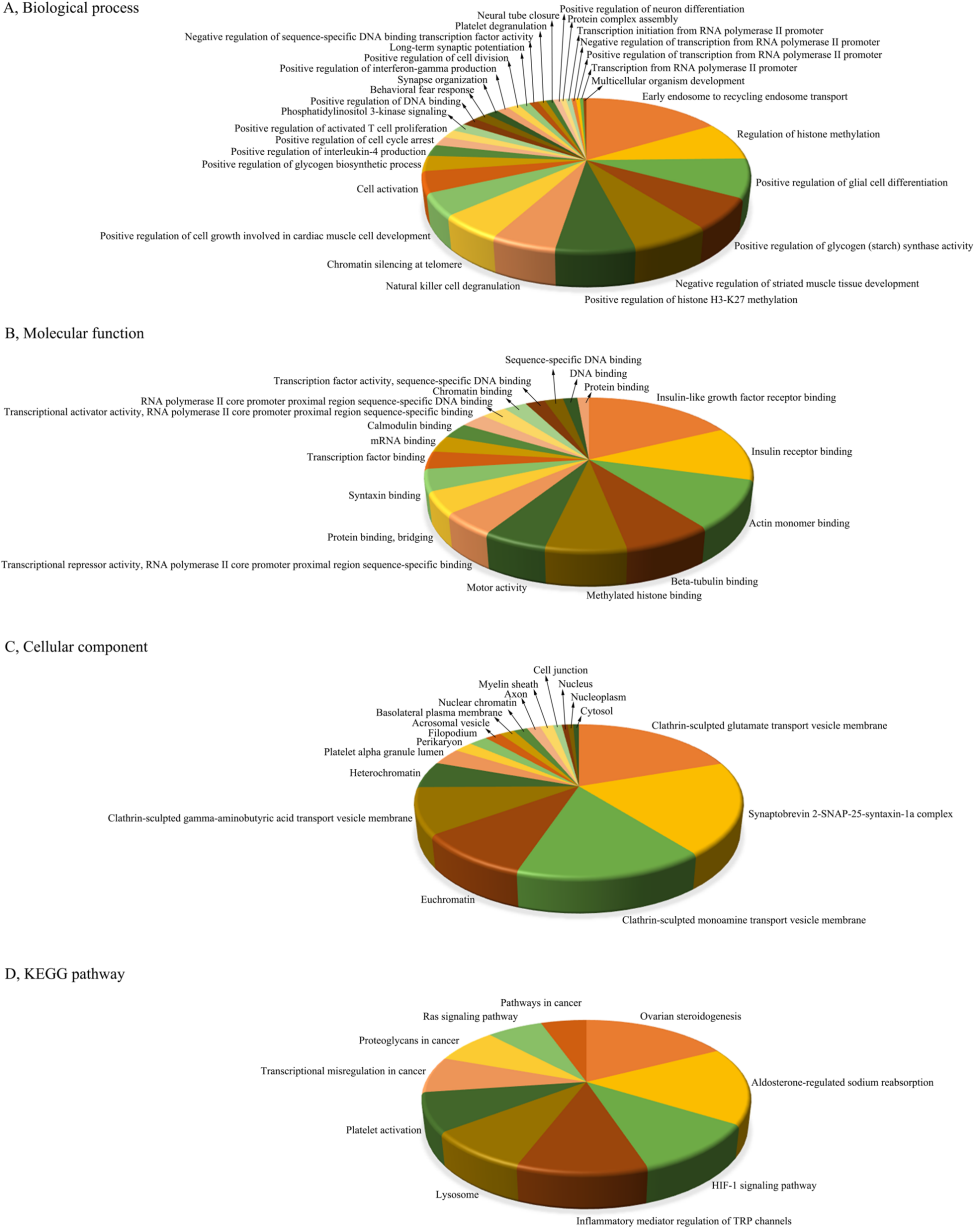
Functional enrichment analysis of target genes of persistent down-regulated miRNAs. **A** GO terms for BP of target genes. **B** GO terms for MF of target genes. **C** GO terms for CC of target genes. **D** KEGG Pathway annotations for target genes

Target genes were analyzed for their potential functions through a gene set enrichment analysis which mainly focused on the GO and KEGG gene set collections. The analysis revealed that the putative target mRNAs of upregulated miRNAs were prone to be found in 68 BPs, 33 MFs, 23 CCs and 14 pathways (Fig. 4), and the putative target mRNAs of downregulated mRNAs contributed to 31 BPs, 19 MFs, 18 CCs and 10 pathways (Fig. 5). Simultaneously, it was obviously seen that these involved GO terms and pathways included some basic biological processes, such as Calcium ion transmembrane transport, Regulation of apoptotic process, cAMP signaling pathway, Signal transduction, etc., and also contained some immune-related mechanisms, such as Positive regulation of interferon-gamma production, Inflammatory mediator regulation of TRP channels, etc., as well as nervous system-related regulation, such as Regulation of axon regeneration, Brain development, Neuroactive ligand-receptor interaction, Positive regulation of neuron differentiation, etc. These results implied that these aberrantly expressed miRNAs have important biological roles in the occurrence and development of HFMD caused by CV-A10.

### The construction of interaction networks

**Fig. 6:**
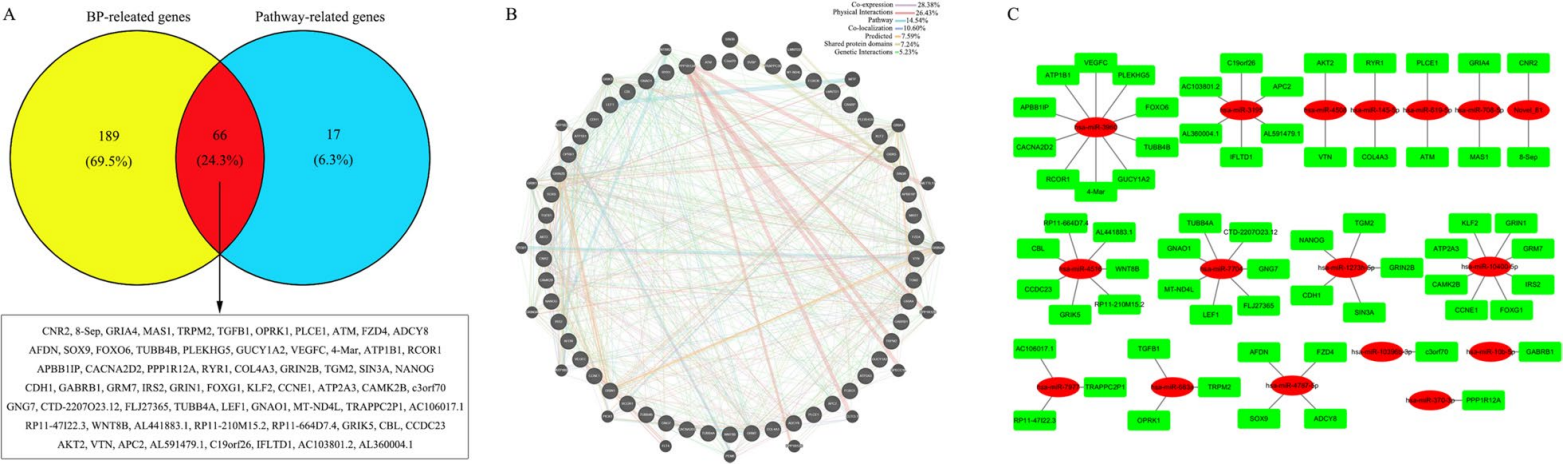
Complex network construction. **A** Venn plot indicating the target genes obtained from the analysis of BP-related genes and Pathway-related genes mediated by up-regulated miRNAs. **B** The co-expression network for the predicted target genes potentially involved in cells-pathogen interaction. **C** Regulatory network linking miRNAs to their putative target genes

**Fig. 7:**
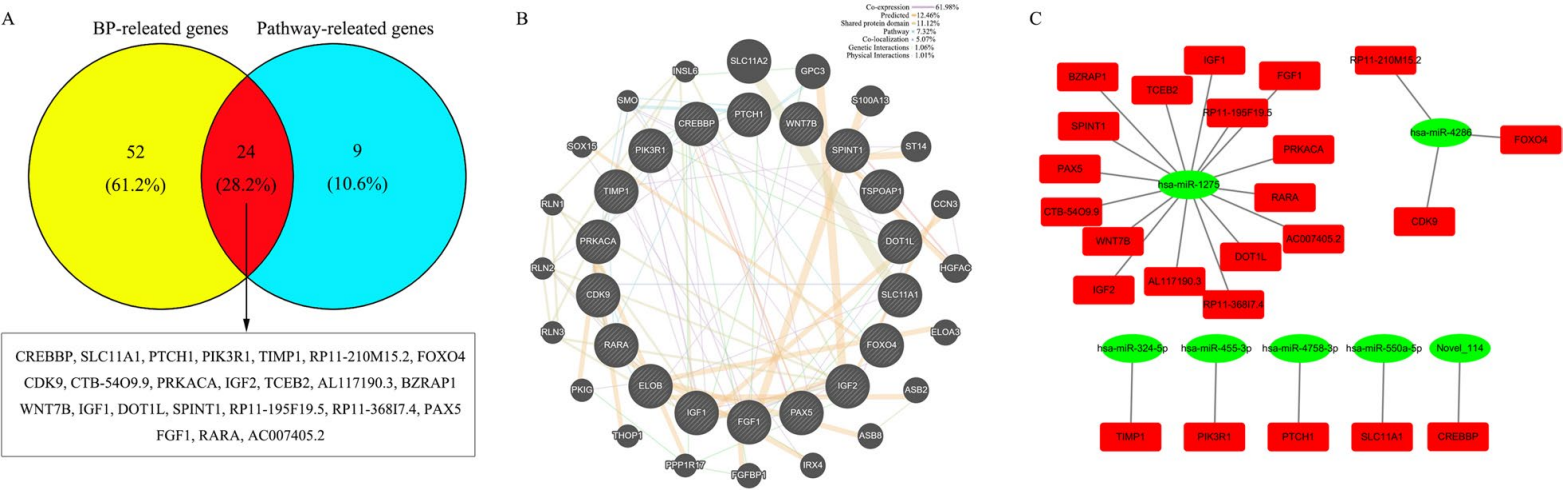
**A** Venn plot indicating the target genes obtained from the analysis of BP-related genes and Pathway-related genes mediated by down-regulated miRNAs. **B** The co-expression network for the predicted target genes potentially involved in cells-pathogen interaction. **C** Regulatory network linking miRNAs to their putative target genes

A Venn diagram was draw to show the intersection between BP-related genes and Pathway-related genes. It was uncovered that there were 66 and 24 target genes detected in the predicted target genes of up- and down-regulated miRNAs, respectively (Figs. 6 and 7A). And then, the establishment of co-expression gene networks based on these screened target genes was performed using GeneMANIA. The co-expression gene network constructed by the 66 target genes from up-regulated miRNAs has 7 main regulative relations, namely Co-expression (28.38%), Physical interaction (26.43%), Pathways (14.54%), Co-localization (10.60%), Predicted (7.56%), Shared protein domains (7.24%) and Genetic interactions (5.23%) (Fig. 6B), but the co-expression gene network constructed by the 24 target genes from down-regulated miRNAs has also 7 main regulative relations, namely Co-expression (61.98%), Predicted (12.46%), Shared protein domains (11.12%), Pathways (7.32%), Co-localization(5.07%), Genetic interactions (1.06%) and Physical interaction (1.01%) (Fig. 7B). Afterwards, we went back to look for miRNAs that regulated these target genes from the data analyzed above and found 17 up-regulated miRNAs and 7 down-regulated miRNAs. Eventually, the miRNA-mRNA regulatory networks were built (Figs. 6 and 7C).

### Confirmation of selected miRNAs and their targets by RT-qPCR

**Fig. 8:**
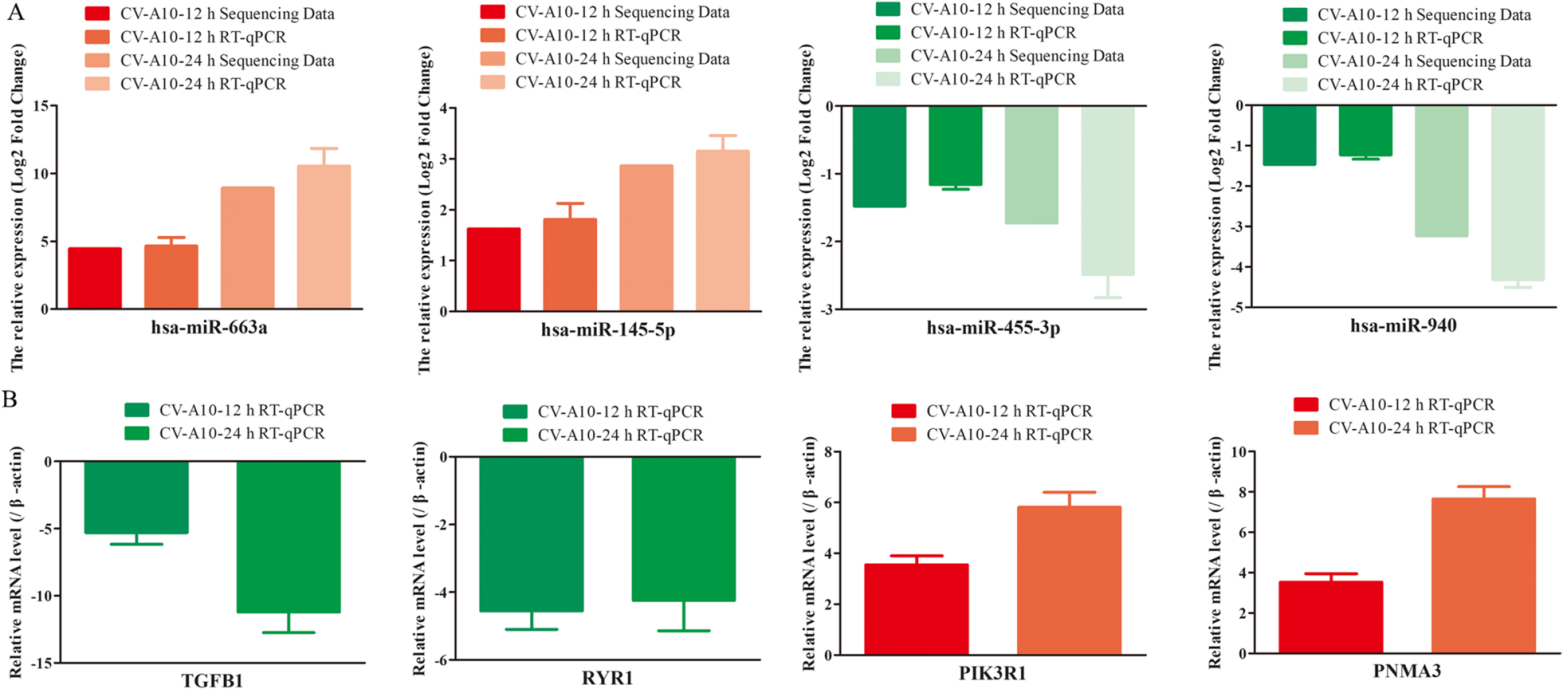
RT-qPCR data showing validation of the sequencing results for differentially regulated miRNAs. **A** and their relevant target genes **B** in HUVEC cells infected with CV-A10

To confirm the reliability and robustness of the sequencing data, 4 miRNAs and their corresponding targets were selected to further assess by RT-qPCR (Table S1). It was observed that compared to control group, the expression levels of hsa-miR-663a and hsa-miR-145-5p were significantly increased, while the expression levels of hsa-miR-455-3p and hsa-miR-940 were significantly decreased in CV-A10-infected groups. All these selected miRNAs showed consistent results with sequencing data (Fig. 8A). Furthermore, the corresponding mRNAs of these selected miRNAs also displayed inverse expressions (Fig. 8B), which was in line with the theory of negative regulation of miRNAs on their target genes.

## Discussion

With the launch and vaccination of EV-A71 vaccine, EV-A71 associated incidence rate and case-severity rate have both decreased, and other serotypes are becoming dominant, especially CV-A10 and CV-A6 [8, 13, 14]. Moreover, CV-A10 is most commonly associated with severe HFMD [13], but there were no preventive vaccines against CV-A10 and no specific therapeutic drugs for CV-A10-associated disease; thereby CV-A10-associated HFMD has become a significant challenge for the prevention and control of HFMD. However, miRNA-mediated regulation of viral infection has been described in a wide variety of hosts and across both DNA and RNA virus families over the past years [8, 22]. Cellular miRNAs directly target host or viral transcripts, which can further viral replication, antiviral immune responses, viral latency, and pathogenesis [8, 28]. Consequently, there has been a large expansion in the number of studies identifying miRNAs as modulating factors during virus infections [29]. For example, miR-548ah promotes the replication and expression of hepatitis B virus (HBV) by targeting histone deacetylase 4 (HDAC4) (PMID: 30,615,846). miR-99a restricts replication of hepatitis C virus (HCV) by targeting mTOR and de novo Lipogenesis [30]. miR-30e-3p inhibits influenza B virus replication by targeting viral NA and NP genes [14]. In recent years, high-throughput sequencing technology has been effectively used to identify differentially expressed miRNAs, on a genome-wide scale, during viral infection [31, 32]. And in our previous studies, we have also identified the differential expression and correlation analysis of miRNA profiles in different type cells infected with EV-A71 and CV-A16 with high-throughput sequencing, which provide some guidance for us to further study the potential pathogenesis of EV-A71 and CV-A16 [22, 25, 33]. In this work, a global miRNA profile was investigated in CV-A10-infected 16HBE cells by high-throughput sequencing approach to determine the differentially expressed miRNAs miRNAs. A total of 258 known and 54 novel differentially expressed miRNAs were successfully identified in CV-A10-12 h group, while a total of 234 known and 44 novel differentially expressed miRNAs were successfully identified in CV-A10-24 h group. The changes in miRNA expression following viral infection were thought to be directly involved in modulating the infection progression of CV-A10. And 4 of these miRNAs were selected for RT-qPCR validation, and they showed similar expression pattern as those revealed by sequencing data, which indicated a low false discovery rate of our sequencing data and supported the credibility of the profile. Meanwhile, based on the negative regulatory relationship of miRNAs to their target regulated genes, we also performed RT-qPCR verification on mRNAs corresponding to these 4 miRNAs. And the results also basically show a relatively consistent result of miRNA negatively regulating mRNA.

To pay more attention to the miRNAs involved in the whole process of CV-A10 infection, the overlapped miRNAs at 12 h and 24 h post-CV-A10 infection were screened and further subjected to unsupervised hierarchical clustering and trend analysis. It was discovered that there were 45 persistent up-regulated and 22 persistent down-regulated during the process of CV-A10 infection, which was considered as the key miRNAs for the following exploration. With the extension of infection time, miRNAs in the same changing trend are considered to be more meaningful. Among these miRNAs, most miRNAs, including let-7e-5p, miR-146a-5p, miR-145a-5p, let-7e-3p and let-7b-3p, etc., have been studied in viral infectious diseases [11]. Moreover, in our previous study, it was verified that miR-4516 was relatively decreased in EV-A71 infection and relatively increased in CV-A16 infection, which uncovered that up-regulation of miR-4516 promoted epithelial barrier injury and miR-4516 contributed to the different functions of epithelial permeability barrier during EV-A17 and CV-A16 infections [34], but in this work, miR-4516 has been found to be up-regulated in CV-A10 infection, which might be a key factor causing epithelial barrier damage during CV-A10 infection that was similar to CV-A16 infection. Subsequently, in order to further insight into the physiological functions and regulatory roles of these persistent changed miRNAs, target genes for these miRNAs were predicted, and enrichment analysis of target genes regulated by these miRNAs was conducted. About 549 and 279 target genes for the up-regulated and down-regulated differentially expressed miRNAs were successfully detected, respectively. Simultaneously, the functional analysis revealed that the putative target genes were are mainly involved in regulation of basic biological processes (such as Calcium ion transmembrane transport, Regulation of apoptotic process, cAMP signaling pathway, Signal transduction, etc.), immune response (such as Positive regulation of interferon-gamma production, Inflammatory mediator regulation of TRP channels, etc.) and nervous system development (such as Regulation of axon regeneration, Brain development, Neuroactive ligand-receptor interaction, Positive regulation of neuron differentiation, etc.), which might be directly linked to pathogenic mechanism of CV-A10 infection. Next, we would discuss the possible significance of some enriched GOs and Pathways after CV-A10 infection. Firstly, apoptosis, having a central influence on viral infection, either assists persistent viral infection or elicits the elimination of infected cells by the host [35]. For example, human herpes simplex virus type 1 (HSV-1) had been shown to interfere with the process of apoptosis in infected cells, which facilitated the establishment and maintenance of persistent infection or prolonging the survival of lytically infected cells [36]; moreover, increasing body of literature has supported that many viruses could utilize host cellular apoptosis to enhance viral replication, such as coxsackievirus B3 (CV-B3), HBV, influenza A virus (IAV) [37]. Moreover, the involvement of miRNAs in virus-mediated apoptosis has been widely studied [38]. For instance, induction of the cellular miR-29c by IAV contributes to virus-mediated apoptosis through repression of antiapoptotic factors BCL2L2 [39]. Thus, the enriched “Regulation of apoptotic process” regulated by differentially expressed miRNAs in the current study might be an important event in virus replication or cell survival after CV-A10 infection. Secondly, viral infections always trigger an immediate immune response in host cells that mainly includes production of a family of cytokines such as interferons (IFNs) and inflammatory cytokines, which interferes with virus replication and affects the viral infection process [40]. For example, the NS1’ protein of JEV is found to antagonize type I IFN production for helping JEV evade antiviral immunity and benefit viral replication [41]; the antiviral mediator IFN-γ, which is elevated in COVID-19, affects epithelial cell differentiation, viral invasion receptor angiotensin-converting enzyme-2 (ACE2) expression, and susceptibility to infection with SARS-CoV-2 and eventually results in replication and transmission of SARS-CoV-2 [42]; severe IAV infections in humans are characterized by excessive inflammation and tissue damage, often leading to fatal disease [43]; in addition, the alterations of aberrant cytokines and chemokines trigger an excessive inflammatory response in EV-A71 infection, which was thought to accelerate the pathogenesis of EV-A71 brain stem encephalitis and pulmonary edema [44]. However, recent evidence has proved that miRNAs have a fundamental role in inducing host’s immune response [45]. For instance, miR-155 regulates the innate immune response by regulating IFN-γ production from natural killer cells during chronic HCV infection, and controls the immune response to HCV in the liver [46]. Thence, the enriched “Positive regulation of interferon-gamma production” and “Inflammatory mediator regulation of TRP channels” regulated by differentially expressed miRNAs in the present study might be the key driver to causing the exacerbation of CV-A10 infection. Thirdly, it is well-known that CV-A10 has caused a series of HFMD outbreaks worldwide, especially in mainland China in recent years and CV-A10 infection has been often reported to be closely related to a high incidence of fatal cardiopulmonary and neurologic complications [5]. However, the underlying mechanism of CNS damage induced by CV-A10 infection remains unclear. Among the enriched GOs and Pathways, there were a lot of nervous system-related regulation, such as “Regulation of axon regeneration”, “Brain development”, “Neuroactive ligand-receptor interaction”, “Positive regulation of neuron differentiation”, etc., which suggested that these might be closely associated with the neuropathogenesis of CV-A10 infection. Conclusively, GO annotation for target genes offer a better understanding of the target genes at biological, molecular and cellular levels [47], whereas KEGG pathway analysis for target genes provide potential signaling pathway transduction mechanisms of the target genes [48]. Accordingly, the above enrichment analysis has ultimately illustrated that the regulatory mechanism of abnormally expressed miRNAs in CV-A10-cell interaction might be largely correlated with the pathogenesis of HFMD caused by CV-A10, especially immune dysregulation and neurological impairment.

In the end, to further explore the roles of key target genes, we focused on genes involved in both BPs and Pathways. Furthermore, the regulatory networks of these genes and their corresponding miRNA were deeply analyzed. First of all, gene co-expression network was established according to their potential inter-relationships. And it was clarified clearly 7 regulative relations, including Co-expression, Physical interaction, Pathways, Co-localization, Predicted, Shared protein domains and Genetic interactions. Afterwards, the dysregulated miRNAs corresponding to these target genes were identified and used to build miRNA-mRNA networks. And based on the theory that miRNAs exert a negatively regulatory role in the gene expressions by targeting the 3’-UTR of mRNAs, this makes it logical to think that miRNAs indirectly participate in the biological processes or pathways involved in their target genes and play a negative regulatory role [10]. Among the miRNA-mRNA network, we arbitrarily picked an up- and a down-regulated miRNA-mediated regulatory axis for discussion, namely the miR-663a-TGFB1 axis and the miR-455-3p-PIK3R1 axis. Actually, the targeted regulatory interaction between miR-663a and TGFB1 has been definitely reported [49]. Furthermore, miR-663a has also been discovered to act as a tumor suppressor in hepatocellular carcinoma [49], renal carcinoma [50], etc. Meanwhile, emerging data have previously confirmed that TGFB1-mediated biological processes or pathways has tumor-suppressor functions in early-stage cancer, including cell-cycle arrest and apoptosis, but can promote tumorigenesis in late-stage cancer, including metastasis and chemoresistance [51], which contributes to the pathogenesis of virus-induced cancers, such as human papillomavirus (HPV) [52], epstein-barr virus (EBV) [53]. Thereby, these findings indicated that the miR-663a-TGFB1 axis might be play a critical role in occurrence and development of virus-induced cancers, but its function on the other viruses infection remains to be investigate. Additionally, miR-455-3p was found to target multiple host genes implicated in the occurrence and development of viral infections. For example, HIV-associated sensory polyneuropathy and neuronal injury are associated with miR-455-3p induction [54]; miR-455-3p was also found to present a differentially expression in H5N1 avian influenza virus infection and hepatitis B virus infection [55, 56]. Meanwhile, previous studies have been confirmed that PIK3R1 have been identified to be differentially expressed in many human cancers and implicated in tumor progression and metastasis [57, 58], but PIK3R1 is increasingly being nominated as a pivotal mediator in the viral infections in recent years. For instance, PIK3R1 was highly expressed in the total T cells of COVID-19 patients, which might lead to defective functions in the T cells and finally determine the pathogenesis of COVID-19 [59]. Although there were no studies have been demonstrated the target interaction between miR-455-3p and PIK3R1, our RT-qPCR vitrificated data have indirectly showed a targeted interaction between miR-455-3p and PIK3R1. Hence, the miR-455-3p-PIK3R1 axis is thought to likely participate in the progression of virus infection. Overall, the analysis of above networks provided a good interpretation of the relationship between miRNA and the relevant target genes, which could further help us elucidate the possible mechanisms of miRNAs in progression of CV-A10 infection.

## Conclusion

This report firstly described miRNA expression profile in 16HBE cells after CV-A10 infection. As a consequence, a large number of dysregulated miRNAs candidates were identified in CV-A10-infected cells compared to uninfected cells, supporting the point that certain miRNAs are essential in host and virus interaction. Then, target prediction and functional analysis showed that these differentially miRNAs involved in various cellular physical process and pathways, suggesting that CV-A10 might affect physical functions by altering host miRNA profiles, thereby prompting virus replication and survival in hosts. Ultimately, the construction of network further revealed the regulatory roles of miRNAs in host-CV-A10 interactions. Collectively, this study makes us gain a comprehensive insight into the contribution of miRNAs to the host-CV-A10 interactions, and also offer new clues to develop auxiliary screening markers or potential therapeutic target for CV-A10 infection.

## Supplementary Information

**Additional file 1**: **Figure S1**. **A**. Infectious virus particles from CV-A10-infected cells were quantitated by virus titer. **B**. The efficacy of CV-A10 infections was measured by an immunofluorescence assay.

**Additional file 2**: **Figure S2**. Typical stem-loop structure of novel differentially expressed miRNAs.

**Additional file 3**: **Table S1**. Selected miRNAs for RT-qPCR.

**Additional file 4**: **Table S2**. Summary of miRNA and mRNA primers used in RT-qPCR.

## Data Availability

All data generated or analyzed during this study are included in this published article.

## Abbreviations

CV-A10: Coxsackievirus A10
HFMD: Hand, foot, and mouth disease
miRNAs: MicroRNAs
EV-A71: Enterovirus 71
CV-A6: Coxsackievirus A6
CV-A10: Coxsackievirus A10
JEV: Japanese encephalitis virus
DENV: Dengue virus
CV-B3: Coxsackievirus B3
CNS: Central nervous system
PBMC: Peripheral blood mononuclear cells
16HBE: Bronchial epithelial cells
HUVECs: Human umbilical vein endothelial cells
GO: Gene ontology
KEGG: Kyoto encyclopedia of genes and genomes
BP: Biological process
MF: Molecular function
CC: Cellular component
HSV-1: Human herpes simplex virus type 1
CV-B3: Coxsackievirus B3
HBV: Hepatitis B virus
IAV: Influenza A virus
IFNs: Interferons
ACE2: Angiotensin-converting enzyme-2
HCV: Hepatitis C virus
HPV: Human papillomavirus
EBV: Epstein-barr virus
