# Supplementary figures and images for: Comprehensive profiling and characterization of cellular microRNAs in response to coxsackievirus A10 infection in bronchial epithelial cells
Source: Virol J. 2022 Jul 21;19:120. doi: 10.1186/s12985-022-01852-9 (PMC9302563; doi:10.1186/s12985-022-01852-9)

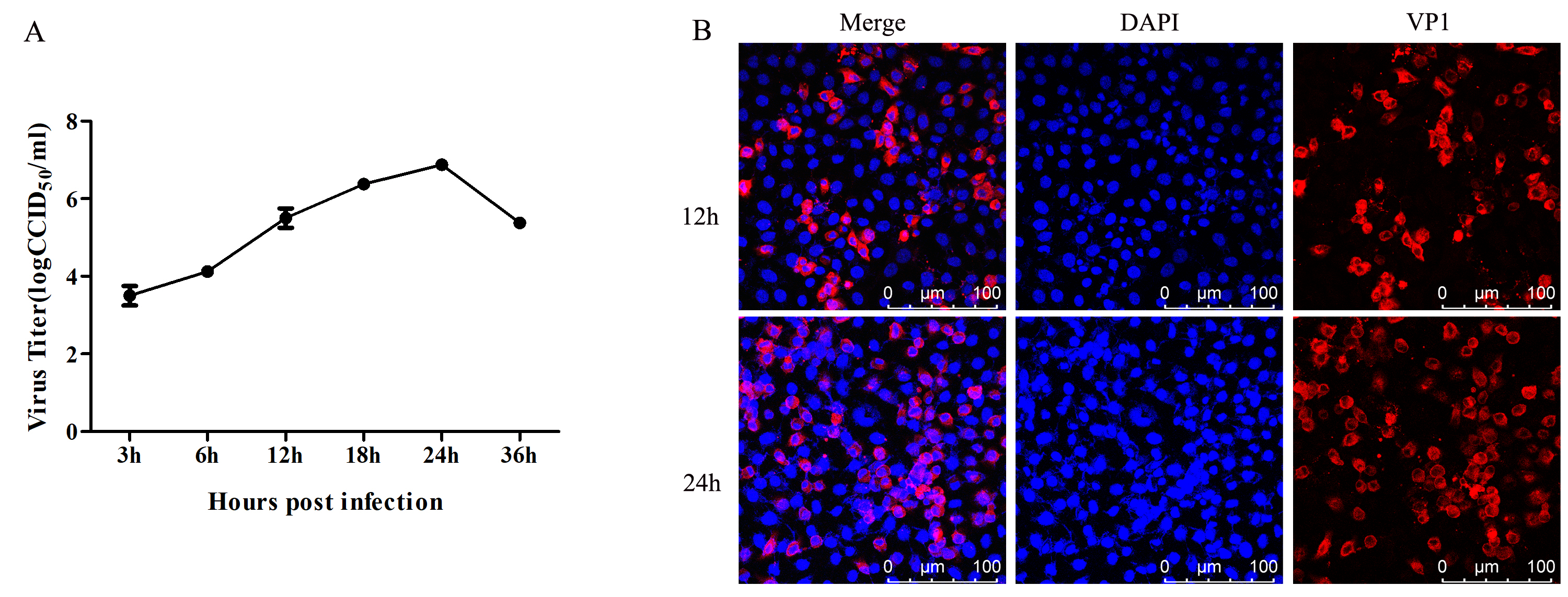

Supplement: Supplementary file 1 — Additional file 1: Figure S1. A. Infectious virus particles from CV-A10-infected cells were quantitated by virus titer. B. The efficacy of CV-A10 infections was measured by an immunofluorescence assay. [file 12985_2022_1852_MOESM1_ESM.tif]

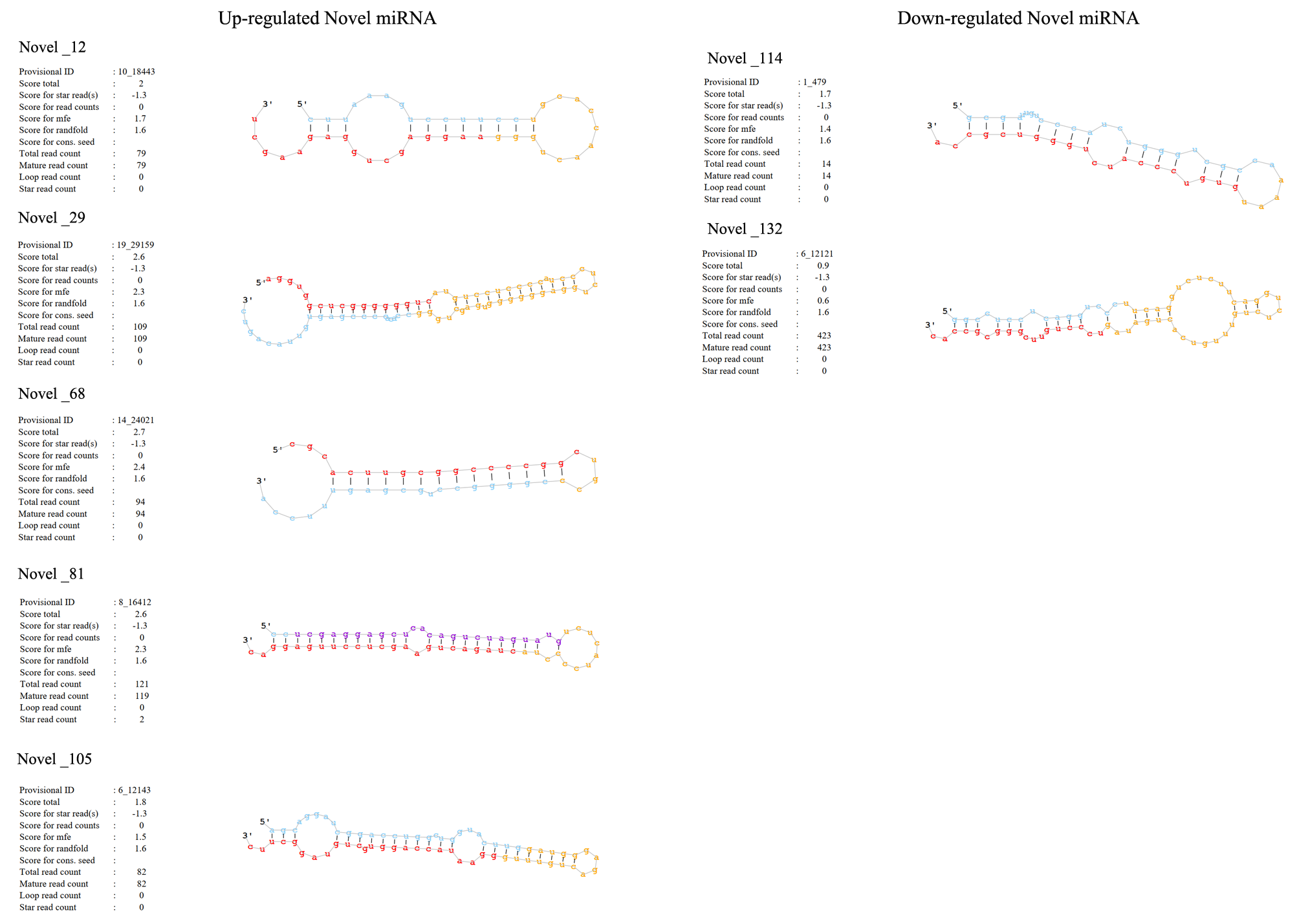

Supplement: Supplementary file 2 — Additional file 2: Figure S2. Typical stem-loop structure of novel differentially expressed miRNAs. [file 12985_2022_1852_MOESM2_ESM.tif]
