# Supplementary material for: Comprehensive profiling and characterization of cellular microRNAs in response to coxsackievirus A10 infection in bronchial epithelial cells
Source: Virol J. 2022 Jul 21;19:120. doi: 10.1186/s12985-022-01852-9 (PMC9302563; doi:10.1186/s12985-022-01852-9)
Supplement: Supplementary file 3 — Additional file 3: Table S1. Selected miRNAs for RT-qPCR. [file 12985_2022_1852_MOESM3_ESM.docx]

**Table S1.** Selected miRNAs for RT-qPCR.

| **miRNA** | **Regulation direction** | **CV-A10-12 h** | **CV-A10-24 h** | **Target genes** |
| --- | --- | --- | --- | --- |
|  |  | **Log_2_FoldChange** | **Log_2_FoldChange** |  |
| hsa-miR-663a | Up | 4.453906746 | 8.933153556 | TGFB1 |
| hsa-miR-145-5p | Up | 1.623831748 | 2.862287225 | RYR1 |
| hsa-miR-455-3p | Down | -1.476304924 | -1.726887527 | PIK3R1 |
| hsa-miR-940 | Down | -1.463631094 | -3.225175616 | PNMA3，BE2Q1 |
