# Supplementary material for: Comprehensive profiling and characterization of cellular microRNAs in response to coxsackievirus A10 infection in bronchial epithelial cells
Source: Virol J. 2022 Jul 21;19:120. doi: 10.1186/s12985-022-01852-9 (PMC9302563; doi:10.1186/s12985-022-01852-9)
Supplement: Supplementary file 4 — Additional file 4: Table S2. Summary of miRNA and mRNA primers used in RT-qPCR. [file 12985_2022_1852_MOESM4_ESM.docx]

**Table S2.** Summary of miRNA and mRNA primers used in RT-qPCR.

| **miRNAs or Target genes** | **Primers** |
| --- | --- |
| hsa-miR-663a | F：5’-GCGCCGCGGGA-3’  R：5’-CCAGTTTTTTTTTTTTTTTGCGGT-3’ |
| hsa-miR-145-5p | F：5’-GTCCAGTTTTCCCAGGAATC-3’  R：5’-AGGTCCAGTTTTTTTTTTTTTTTAGG-3’ |
| hsa-miR-455-3p | F：5’-GGCAGTCCATGGGCAT-3’  R：5’-GGTCCAGTTTTTTTTTTTTTTTGTGT-3’ |
| hsa-miR-940 | F：5’-GGGCCCCCGCT-3’  R：5’-CCAGTTTTTTTTTTTTTTTGGGGA-3’ |
| TGFB1 | F：5’-TACCTGAACCCGTGTTGCTCT-3’  R：5’-CTGCCGCACAACTCCGGTGA-3’ |
| RYR1 | F：5’-ACCGCCTAAATGTCTACACCAC-3’  R：5’-TTGCTACGATTGCCACGGAT-3’ |
| PIK3R1 | F：5’-GAATATACCCGCACATCCCA-3’  R：5’-TTTCTCATTGCCTTCACGTT-3’ |
| PNMA3 | F：5’-TTGTGTTACGTTTGGAACCCCT-3’  R：5’-TGGCCCCACTTAAGACTCGTT-3’ |
